# Unidirectional Flux Balance of Monovalent Ions in Cells with Na/Na and Li/Na Exchange: Experimental and Computational Studies on Lymphoid U937 Cells

**DOI:** 10.1371/journal.pone.0153284

**Published:** 2016-05-09

**Authors:** Igor A. Vereninov, Valentina E. Yurinskaya, Michael A. Model, Alexey A. Vereninov

**Affiliations:** 1 Peter the Great St-Petersburg Polytechnic University, St-Petersburg, Russia; 2 Laboratory of Cell Physiology, Institute of Cytology, Russian Academy of Sciences, St-Petersburg, Russia; 3 Department of Biological Sciences, Kent State University, Kent, Ohio, 44242, United States of America; Glasgow University, UNITED KINGDOM

## Abstract

Monovalent ion traffic across the cell membrane occurs via various pathways. Evaluation of individual fluxes in whole cell is hampered by their strong interdependence. This difficulty can be overcome by computational analysis of the whole cell flux balance. However, the previous computational studies disregarded ion movement of the self-exchange type. We have taken this exchange into account. The developed software allows determination of unidirectional fluxes of all monovalent ions via the major pathways both under the balanced state and during transient processes. We show how the problem of finding the rate coefficients can be solved by measurement of monovalent ion concentrations and some of the fluxes. Interdependence of fluxes due to the mandatory conditions of electroneutrality and osmotic balance and due to specific effects can be discriminated, enabling one to identify specific changes in ion transfer machinery under varied conditions. To test the effectiveness of the developed approach we made use of the fact that Li/Na exchange is known to be an analogue of the coupled Na/Na exchange. Thus, we compared the predicted and experimental data obtained on U937 cells under varied Li^+^ concentrations and following inhibition of the sodium pump with ouabain. We found that the coupled Na/Na exchange in U937 cells comprises a significant portion of the entire Na^+^ turnover. The data showed that the loading of the sodium pump by Li/Na exchange involved in the secondary active Li^+^ transport at 1–10 mM external Li^+^ is small. This result may be extrapolated to similar Li^+^ and Na^+^ flux relationships in erythrocytes and other cells in patients treated with Li^+^ in therapeutic doses. The developed computational approach is applicable for studying various cells and can be useful in education for demonstrating the effects of individual transporters and channels on ion gradients, cell water content and membrane potential.

## Introduction

The concept of the pump-leak flux balance as the basis of monovalent ion gradients at the animal cell membrane is universally accepted. A number of various transporters and channels are involved in continuous ion traffic across the membrane and many of them are capable of passing ions both inward and outward. However, discrimination between fluxes via specific ways is not a trivial problem because any macroscopic ion transfer is accompanied by disturbance of cell water and electrical balance. Fluxes of different ions and via different routes appear to be interdependent due to the mandatory conditions of electroneutrality and osmotic balance. In addition, some transporters operate as a co- or counter-transporters. Calculation of the overall flux balance and prediction of its dependence on specific properties of transporters and channels can be performed by the computational solution of a set of nonlinear differential equations [1–9]. However, there are no sufficiently simple computational tools for solution of real cell physiology problems. Most experimentalists continue to neglect computational approaches because many parameters are required for modeling, whose evaluation is difficult and unreliable. Not all types of the monovalent ion movement across the cell membrane are accounted for in the available models. Ion traffic of the self-exchange type that comprises a significant portion of Na^+^ and Cl^−^fluxes across the membrane remained beyond the scope of previous models. We aimed to develop relatively simple software for analyzing the effects of various transporters and channels on cell water-volume, membrane potential and related cell properties under various conditions suitable for researchers with limited programming expertise.

Our approach was developed initially for studying Li^+^ transport. Li^+^ is the closest physiological analogue of Na^+^ and the Li/Na exchange is the closest analogue of the Na/Na exchange. Li^+^ is a poor substrate for the Na/K-ATPase pump but it passes through the same channels as Na^+^, and their gradients on the cell membrane are comparable. For example, the ratio of balanced intracellular to extracellular concentrations in U937 cells is 0.82–0.96 for Li^+^ and 0.28–0.30 for Na^+^, whereas for K^+^ it is 30–32 [10]. It is the Li/Na exchange that mediates secondary active Li^+^ transport out of cells [10–13]. The mechanism of Li^+^ transport and of Li/Na exchange, in particular, is important for a number of practical reasons: alteration of Li/Na exchange in erythrocytes accompanies widespread human pathologies (hypertension, diabetes, nephropathy etc.); Li^+^ is used as a medication for treatment of neuropsychiatric disorders and testing renal clearance [10, 14–18].

## Materials and Methods

U937 human histiocytic lymphoma cells were obtained from the Russian Cell Culture Collection (cat. number 160B2). The cells were cultured in RPMI 1640 medium (Biolot, Russia) with 10% fetal calf serum (HyClone, USA). Ouabain and dimethylamiloride (DMA) were purchased from Sigma-Aldrich (Germany), Percoll was from Pharmacia (Sweden) and the salts (all of analytical grade) were from Reachem (Russia). Intracellular cation content was determined by flame emission on a Perkin-Elmer AA 306 spectrophotometer, Cl^−^content by distribution of ^36^Cl^−^(Isotope, Russia) and chemical external Cl^−^assay, cell water was evaluated by cell buoyant density in Percoll density gradient, and protein was measured by the Lowry method. The experimental methods used in this work have been described in detail earlier [10, 19–24]. Na^+^ and Cl^−^fluxes were estimated using ^22^Na^+^ and ^36^Cl^–^. For quantifying the pump-mediated Rb^+^ influx cells were incubated in the medium with 2.5 mM RbCl with and without 0.1 mM ouabain for 10 min. To study ion efflux, cells were pre-equilibrated in the presence of ions for either 1.5 h (with 5 mM LiCl, ^22^Na^+^ or ^36^ Cl^–^) or 20 h (with 1 mM RbCl). Next, the cells were rapidly washed with 96 mM MgCl_2_ and placed into fresh RPMI medium. The radioactivity of ^36^Cl‾ was analyzed using a Beckman LS 6500 liquid scintillation counter, and radioactivity of ^22^Na^+^ was counted using a Na-Iodide detector. The equilibration rate coefficients *k* were calculated according to *y*_*t*_ *= y*_*∞*_(1– exp (*-kt*)) for influx and *y*_*t*_ *= y*_*0*_ exp (*-kt*) for efflux, where *y*_*t*_ is the content of ion-label at time *t* (5, 10, 20 min) and *y*_*∞*_ and *y*_*0*_ are the final and initial content of the corresponding ion. Li^+^ balanced distribution in U937 cells was studied at 1–10 mM LiCl added to the culture medium. To replace intracellular K^+^ and Na^+^ with Li^+^, cells were incubated in a medium containing 140 mM LiCl, 5.5 mM KCl, 0.42 mM CaCl_2_, 0.41 mM MgCl_2_, 10 mM Hepes, pH 7.4 was adjusted with LiOH. To minimize errors in determination of the Li/Na discrimination coefficients, the medium was analyzed simultaneously with the cell samples. The standard value of 140 mM for Na^+^ concentration in RPMI medium was used in other calculations.

The results were analyzed using Student’s t-test. Differences were considered significant at P<0.05.

## Results

### Overview of the movement and distribution of monovalent ions in U937 cells

**Fig 1:**
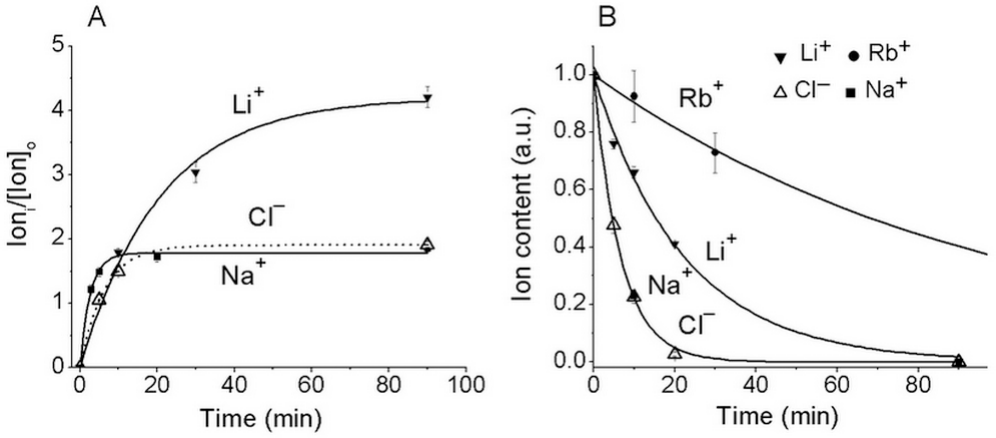
**Kinetics of gain (A) and loss (B) of ions in U937 cells.** The measurements were performed as described in Methods. (A) Ratio of intracellular ion content Ion_i_ (μmol per g protein) to its concentration in the medium [Ion]_o_ (mM); (B) Ion content in relative units. Symbols stand for means ± SE for 3–4 experiments with 2–3 parallels; solid lines show the best fit based on monoexponential kinetics.

**Fig 2:**
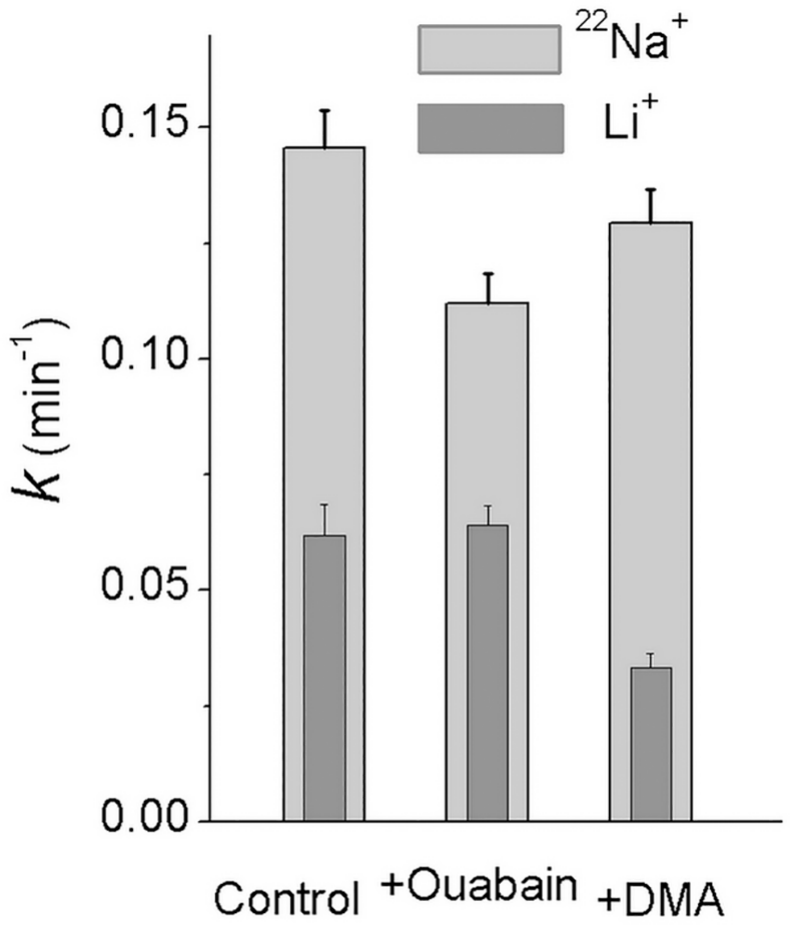
Rate coefficients for ^22^Na^+^ and Li^+^ exit out of U937 cells with and without 0.1 mM ouabain or 0.05 mM DMA. Means ± SE for 9 experiments with two parallels in each are presented.

The kinetics of gain and loss of monovalent ion tracers is shown in Fig 1. The rate of equilibration is the highest for Cl^−^and Na^+^, intermediate for Li^+^, and the lowest for Rb^+^, which is a known analogue of K^+^. By contrast, with Na^+^, the rate coefficient for Li^+^ exit is not reduced in the presence of ouabain (Fig 2). This agrees with the notion that the Na/K ATPase pump is not involved in Li^+^ efflux out of cell. The efflux of Li^+^ is reduced by about two-fold by DMA, an inhibitor of the Na/H exchanger. Intracellular Na^+^ and Li^+^ concentrations in U937 cells equilibrated with 1–10 mM external Li^+^ are presented below. The rates of ion tracer equilibration, as in Fig 1, correspond to uptake and loss of about 15% of cell Na^+^ and Cl^–^, 5% of Li^+^, and 1% of Rb^+^ (K^+^) content per min. The corresponding data in absolute units for the U937 subline 160B2 cells [10, 19, 22, 24] were 30–40 μmol^-1^min^-1^ per g protein for Na^+^ and Cl^−^and 6–7 μmol^-1^min^-1^ for K^+^.

### Computational dissection of ion traffic via specific routes and determination of parameters characterizing the cell as a dynamic electrochemical system

The mathematical model of monovalent ion traffic across the plasma membrane was similar to the one used by Jakobsson [3] and Lew et al. [4–6, 25]. It accounts for the Na/K pump, electroconductive channels, cotransporters NC, NKCC, KC, and a Na/Li countertransporter similar to the Na/H [6]. In this approach, the entire multiplicity of ion transfer systems is replaced by a reduced number of ion pathways defined thermodynamically. All the major pathways are subdivided into five subtypes by ion-driving force: ion channels, where the driving force is the transmembrane electrochemical potential difference for a single ion species; NKCC, NC and KC cotransporters, where the force is the sum of the electrochemical potential differences for all partners; and the Na/K ATPase pump, where ion movement against electrochemical gradient is energized by ATP hydrolysis. This allows characterization of intrinsic properties of each pathway by a single rate coefficient. The following abbreviations are used to designate ion transporters: NKCC (LKCC) indicates the known cotransporters of the SLC12 family carrying monovalent ions with stoichiometry 1Na^+^(Li^+^):1K^+^:2Cl^-^ and KC and NC (LC) stand for cotransporters with stoichiometry 1K^+^:1Cl- or 1Na^+^(Li^+^):1Cl^-^. The latter can be represented by a single protein, the thiazide-sensitive Na-Cl cotransporter (SLC12 family), or by coordinated operation of the exchangers Na/H (SLC9) and Cl/HCO3 (SLC26). Monovalent ions are the main factor in maintaining electrical and water balance in animal cells. Because concentration of free Ca^++^ and Mg^++^ in the cytoplasm is low; their impact on electrical and osmotic balance, as well as on the transmembrane flux balance, is small, and these ions were not included in the calculations. Numerical integration of the flux equations was carried out as described in the Appendix. The variables and parameters used in the software are listed in Table 1. Some of the data necessary for the initial file DATAP.txt (Table 2) were obtained experimentally; these are the intra- and extracellular monovalent ion concentrations, extracellular concentration of membrane-impermeant osmolytes (*B0*), the ratio of ouabain-sensitive to ouabain-resistant Rb^+^ (K^+^) influx (OSOR) and the Na/K pump rate coefficient *β*, which is calculated from the measured [Na]_i_ and ouabain-sensitive Rb^+^ influx. The process of finding the rate coefficients characterizing the ion pathways–*pna*, *pk*, *pl*, *pcl*, *inc*, *ikc*, *ilc*, *inkcc*, *ilkcc* is discussed below.

**Table 1:** Symbols and definitions.

Definitions	In text, Figures and Equations	In files DATAP, RESP	Units
Ion species	Na^+^, Li^+^, K^+^, Cl^–^, Rb^+^	Na, K, Li, Cl	
Types of cotransport	NC, NKCC, KC, LC, LKCC		
Concentration of ions in cell water or external medium	[Na]_i_, [K]_i_, [Li]_i_, [Cl]_i_ [Na]_o_, [K]_o_, [Li]_o_, [Cl]_o_	*na*, *k*, *l*, *cl*, *na0*, *k0*, *l0*, *cl0*	mM
External concentration of membrane-impermeant osmolytes	[B]_o_	*B0*	mM
Intracellular content of membrane-impermeant osmolytes	*A*		mmol, may be related to g cell protein or cell number etc.
Cell water volume	*V*		ml, may be related to g cell protein or cell number etc.
Membrane-impermeant osmolyte concentration in cell water	*A/V*	*A/V*	mM
Cell water content per unit of A	*V/A* x1000	*V/A* x 1000	ml·mmol^-1^
Mean valence of membrane-impermeant osmolytes, A	*z*	*z*	dimensionless
Permeability coefficients	*p*_*Na*_, *p*_*K*_, *p*_*Cl*_, *p*_*Li*_	*pna*, *pk*, *pcl*, *pl*	min^-1^
Na/K pump rate coefficient	*β*	*beta*	min^-1^
Li/K pump rate coefficient	*α*	*alpha*	min^-1^
Na/K, Li/K pump fluxes stoichiometry	*γ*	*gamma*	dimensionless
Membrane potential, MP	*U*	*U*	mV
Dimensionless membrane potential	*u*, *U = uRT*/F	*u*	dimensionless
Net fluxes mediated by cotransport NC, NKCC, KC, LC, LKCC, and Li/Na countertransport LN	*J*_*NC*_, *J*_*NKCC*_, *J*_*KC*_, *J*_*LC*_, *J*_*LKCC*_, *J*_*LN*_	*NC*, *NKCC*, *KC*, *LC*, *LKCC*, *LN*	μmol·min^-1^·(ml cell water)^-1^
Na efflux via the pump	*-β[Na]*_*i*_	*PUMPN (Na)*	μmol·min^-1^·(ml cell water)^-1^
Li efflux via the pump	*-α[Li]*_*i*_	*PUMPL (Li)*	μmol·min^-1^·(ml cell water)^-1^
K influx via the pump	*β[Na]*_*i*_*/γ*	*PUMPN (K)*	μmol·min^-1^·(ml cell water)^-1^
	*α[Li]*_*i*_*/γ*	*PUMPL (K)*	μmol·min^-1^·(ml cell water)^-1^
Net fluxes mediated by channels		*Channel*	μmol·min^-1^·(ml cell water)^-1^
Unidirectional influxes of Na, K, Li or Cl via channels, cotransport NC, KC, LC, NKCC, LKCC or countertransport LN		*IChannel*, *INC*, *IKC*, *ILC*, *ILN*, *INKCC*, *ILKCC*	μmol·min^-1^·(ml cell water)^-1^
Unidirectional effluxes of Na, K, Li or Cl via channels, cotransport NC, KC, LC, NKCC, LKCC or countertransport LN		*EChannel*, *ENC*, *EKC*, *ELC*, *ELN*, *ENKCC*, *ELKCC*	μmol·min^-1^·(ml cell water)^-1^
Time derivatives of concentrations		*prna*, *prk*, *prl*, *prcl*	mM·min^-1^
Co- and counter-transport rate coefficients	*i*_*NC*_, *i*_*KC*_, *i*_*LC*_, *k*_*P*_	*inc*, *ikc*, *ilc*, *kp*	ml·μmol^-1^·min^-1^
	*i*_*NKCC*_, *i*_*LKCC*_	*inkcc*, *ilkcc*	ml^3^·μmol^-3^·min^-1^
“Old” and “new” external concentration of all osmolytes	*S*_*oO*_, *S*_*oN*_,		mM
S_oN_/S_oO_ ratio	*kv*	*kv*	dimensionless
Number of time points between output of results		*hp*	dimensionless
Ratio of ouabain-sensitive to ouabain-resistant Rb^+^ (K^+^) influx	*OSOR*	*OSOR*	dimensionless
Li/Na discrimination coefficient, ([Li]_i_ /[Na]_i_)·([Na]_o_/[Li]_o_)	*c*_*d*_		dimensionless

**Table 2:** The layout of the file DATAP.txt.

*na0*	*k0*	*cl0*	*l0*	*B0*	*kv*	*na*	*k*	*l*	*cl*	*alpha*	*beta*	*gamma*	*pna*	*pk*
140	5.8	121	5	48.2	1.032	37	158	0.0001	63	0	0.039	1.5	0.00349	0.0229
*pl*		*pcl*		*inc*		*ikc*	*ilc*		*inkcc*		*ilkcc*	*kp*	*hp*	
0.00349		0.00426		0.00003		0.0	0.00018		0.0		0.0	0.0002	500	

The parameters *na0*, *k0*, *l0*, *cl0* and *B0* are extracellular ion concentrations (mM); *na*, *k*, *l* and *cl* are intracellular concentrations; *kv* is the ratio of the external to initial internal osmolarity; *alpha* and *beta* are the rate coefficients (min^-1^) for Li/K and Na/K pumps, respectively; *gamma* is the pump Na/K stoichiometric coefficient; *pna*, *pk*, *pl*, *pcl* are the channel permeability coefficients (min^-1^); *inc*, *ikc*, *ilc*, *inkcc*, *ilkcc* are the rate coefficients for the NC, KC, LC (ml·μmol^-1^·min^-1^) and for NKCC, LKCC (ml^3^·μmol^-3^·min^-1^) cotransporters; *kp* is the rate coefficient for Li/Na countertransport. The parameter *hp* is the number of integration steps between data output and corresponds to the real time of the transient process in min. If one wishes to follow the initial stages of the process, *hp* should be made small (5–50), and if the process is slow and one is interested in the balanced state, *hp* can be increased to 1000–5000. The data from a typical experiment with U937 cells are used in this example.

The executable file LIP.exe generates a file RESP.txt which shows the evolution of the cell toward the balanced state, when the influx and efflux of every ion species become equal (Table 3). The fluxes via the major pathways are given for the last state. In some cases the balanced state cannot be reached, e.g. when the conditions of macroscopic electroneutrality and osmotic balance are not fulfilled or the membrane potential falls outside the range +5 mV to –173 mV (computer reports RANGE LIMIT). One should keep in mind that the intracellular concentrations [Na]_i_, [K]_i_, [Cl]_i_, [Li]_i_ and the total extracellular concentration of osmolytes define the important intrinsic cell parameter *z* (the mean valence of membrane-impermeant intracellular osmolytes). Therefore, the balanced states calculated for different initial [Na]_i_, [K]_i_, [Cl]_i_, [Li]_i_ may be different even when the composition of the medium and all rate coefficients are identical.

**Table 3:** Transition of the system to the balanced state as displayed in the file RESP.txt.

**A** Parameter values														
*na0*	*k0*	*cl0*	*l0*	*B0*	*kv*	*alpha*	*beta*	*gamma*	*z*	*kp*	*hp*			
140	5.8	121	5	48.2	1.03	0.000	0.039	1.5	-2.53	0.0002	500			
*pna*	*pk*	*pl*	*pcl*	*inc*	*ikc*	*ilc*	*inkcc*	*ilkcc*	*A/V*					
0.00349	0.0229	0.00349	0.00426	3E-5	0	0.00018	0	0	49.76				
**B** Time course of variables														
*t*	*U*	*na*	*k*	*l*	*cl*	(*V/A*)x*1000*	*mun*	*muk*	*mul*	*mucl*	*prna*	*prk*	*prl*	*prcl*
0	-49.4	38.2	163.1	0.000	65	18.61	-84.1	39.7	-337.4	32.8	0.0000	0.000	0.0000	0.000
50	-48.5	38	158.4	3.882	67.3	19.06	-83.3	39.8	-55.2	32.8	0.0197	0.056	0.0231	0.031
^**^														
450	-47.6	38.6	155.3	4.379	71.9	20.06	-82	40.2	-51.1	33.7	0.0003	0.001	0.0001	0.004
500	-47.5	38.6	155.2	4.375	72.1	20.10	-81.9	40.2	-51.1	33.7	0.0002	0.001	0.0001	0.003
**C** Flux balance under the balanced state														
*Net flux*	*PUMPN*	*PUMPL*	*Channel*	*NC*	*LC*	*LN*	*KC*	*NKCC*	*LKCC*					
Na	-1.5051	0.0000	0.9975	0.4248	0.0000	0.0839	0.0000	0.0000	0.0000					
K	1.0034	0.0000	-0.9989	0.0000	0.0000	0.0000	0.0000	0.0000	0.0000					
Li	0.0000	0.0000	0.0319	0.0000	0.0521	-0.0839	0.0000	0.0000	0.0000					
Cl	0.0000	0.0000	-0.4712	0.4248	0.0521	0.0000	0.0000	0.0000	0.0000					
*Influx*	*PUMPN*	*PUMPL*	*IChannel*	*INC*	*ILC*	*ILN*	*IKC*	*INKCC*	*ILKCC*					
Na	0.0000	0.0000	1.0462	0.5082	0.0000	0.1225	0.0000	0.0000	0.0000					
K	1.0034	0.0000	0.2844	0.0000	0.0000	0.0000	0.0000	0.0000	0.0000					
Li	0.0000	0.0000	0.0374	0.0000	0.1089	0.0386	0.0000	0.0000	0.0000					
Cl	0.0000	0.0000	0.1861	0.5082	0.1089	0.0000	0.0000	0.0000	0.0000					
*Efflux*	*PUMPN*	*PUMPL*	*EChannel*	*ENC*	*ELC*	*ELN*	*EKC*	*ENKCC*	*ELKCC*					
Na	-1.5051	0.0000	-0.0486	-0.083	0.0000	-0.0386	0.0000	0.0000	0.0000					
K	0.0000	0.0000	-1.2833	0.0000	0.0000	0.0000	0.0000	0.0000	0.0000					
Li	0.0000	0.0000	-0.0055	0.0000	-0.057	-0.1225	0.0000	0.0000	0.0000					
Cl	0.0000	0.0000	-0.6573	-0.083	-0.057	0.0000	0.0000	0.0000	0.0000					
*OSOR*	3.53													

** not shown time points

Our executable file allows the solution of the forward problem–finding the intracellular concentrations [Na]_i_, [K]_i_, [Cl]_i_, **[**Li]_i_, cell water content, membrane potential *U*, OSOR, etc. corresponding to the balanced state for a set of parameters given in Table 2. Those values can be compared with experimental data as a test that the parameters have been chosen correctly. The inverse problem (finding the parameters from experimental data) is much harder. There are mathematical methods [26] and software packages [27] for finding optimal parameters in similar cases. However, the use of automated procedures requires more mathematical sophistication than a simple trial and error approach, as well as consideration of specific properties of the system. Most importantly, the system of equations for describing cell ion balance has only a unique solution for a balanced state within the acceptable range of variables as can be verified by using our soft. It should be noted that the accuracy of parameter estimation depends on the dispersion of the experimental data and not on the numerical solution procedure, because the calculation process continues until the difference between the calculated and experimental data becomes less than the experimental error. In the case of U937 cells the parameters *ikc*, *inkcc*, *ilkcc* can be excluded for the following reasons. The inside Cl^−^electrochemical potential under the normal balanced state significantly differs from that outside (*mucl = +*33.7 mV, Table 3). Therefore, a secondary active transport of Cl^−^into the cells should be present. The driving force for Cl^−^ions involved in cotransport is determined by the sum of the electrochemical potential differences for all the partners. Under normal conditions, these sums for NC, KC and NKCC pathways in U937 cells are –48.2, +73.9 and +25.7 mV, respectively (Table 3, *mun+mucl*, *muk+mucl*, *mun+muk+2mucl*). Therefore, the net Cl^−^flux should be directed outward through KC and NKCC and inward through NC. As the integral Cl^−^transport, which creates the +33.7 mV net electrochemical potential difference for Cl^–^, is directed into the cell, the net fluxes through the KC and NKCC routes have to be negligibly small in comparison with the NC cotransport. The small NKCC and KC impact is confirmed by the fact that the effects of bumetanide and DIOA, used as inhibitors of NKCC and KC pathways, are small in the U937 cells compared to epithelial cells [20]. It is reasonable to accept that permeability of electroconductive channels for Li^+^ and for Na^+^ are similar, i.e. *pl* ≈ *pna* [28]. The channel permeability coefficients *pna*, *pk*, *pl*, *pcl* were found by trial and error by seeking the same balanced state as was observed in the experiment. This is not a particularly time-consuming procedure if one takes into account that [K]_i_ and [Na]_i_ are affected mostly by the parameter ratios *pna*/*β* and *pk*/*β*, while [Cl]_i_ and cell water content per unit of impermeant intracellular osmolyte *A* are mostly affected by *inc*/*β* and *pcl*/*β* [9]. Analysis of the present model shows that [Li]_i_ depends mostly on *ilc*/*β*, *pl*/*β* and *kp*.

**Fig 3:**
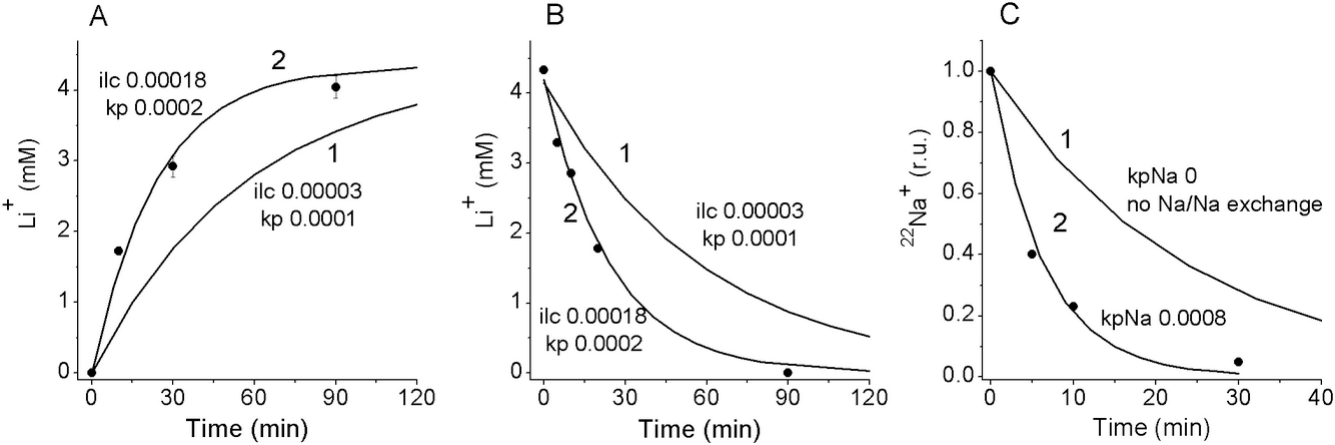
Experimental (symbols) and computed (lines) kinetics of Li^+^ and Na^+^ gain and exit. The values *ilc* and *kp* used in computation are indicated in the graphs. The other parameters are as in Table 2.

Computation shows that the balanced intracellular Li^+^ concentration of 4.37 mM, as well as the other variables observed in U937 cells upon addition of 5 mM LiCl, could be obtained at many combinations of *ilc* and *kp*. To specify *ilc* and *kp*, kinetic data on Li^+^ equilibration were used. Only a unique pair of *ilc* and *kp* (*ilc* = 0.00018, *kp* = 0.0002) corresponds to the real kinetics of Li^+^ gain and exit. Curves 1 in Fig 3A and 3B show the results obtained at values of *ilc* and *kp* that fit the observed balanced state but not the transition kinetics, whereas curves 2 fit both.

Table 3 imitates the table displayed when the calculation is finished. In addition to the initial parameters already contained in Table 2, the parameters and variables in Table 3 include the following: current intracellular ion concentrations *na*, *k*, *l*, *cl* (mM); membrane potential *U* (mV); cell water content *V/A* x *1000* (ml per mmol of *A*); transmembrane electrochemical potential differences *mun*, *muk*, *mul*, *mucl* (mV) for Na^+^, K^+^, Li^+^ and Cl^–^, respectively, calculated as *mun* = 26.7·ln(*na*/*na0*)+*U*, *muk* = 26.7·ln(*k*/*k0*)+*U*, *mul* = 26.7·ln(*l*/*l0*)+*U*, *mucl =* 26.7·ln(cl/cl0)–*U*; the derivatives *prna*, *prk*, *prl*, *prcl*, characterizing the rate of change in [Na]_i_, [K]_i_, [Li]_i_ and [Cl]_i_; net fluxes of Na^+^, K^+^, Li^+^ and Cl^−^via separate ion pathways (μmol·ml^-1^·min^-1^); *A/V* (mM) and the mean valence of membrane-impermeant intracellular osmolytes, *z*. The displayed values of fluxes, as well as *OSOR*, the ratio of ouabain-sensitive to ouabain-resistant K^+^ influx, are calculated only in the bottom line, i.e. for the latest time point. The fluxes at any time can be found by varying *hp*. The values of fluxes per g of cell protein can be found by multiplying the displayed values by cell water content per g of cell protein, which is close to 6.5 ml·g^-1^ in “standard” U937 cells under normal conditions. Transmembrane electrochemical potential differences *mun*, *muk*, *mul*, *mucl* expressed in mV can be converted to J/mol as 1 mV = 10^-3^F ^-1^ (J/mol).

### Validation of the model by studying Li^+^ balanced distribution at 1–10 mM LiCl

We showed earlier that the presence of 3–5 mM Li^+^ in the medium for 12–24 h has no effect on cell proliferation, cell morphology or the balance of monovalent ions in U937 cells [10]. Table 4 presents experimental data on Li^+^ balanced distribution in U937 cells incubated in RPMI medium with addition of 1–10 mM LiCl. The ratio of balanced intracellular to extracellular concentrations were 0.82–0.96 for Li^+^, 0.28–0.30 for Na^+^, and 30–32 for K^+^. These results confirm that the distributions of Li^+^ and Na^+^ are qualitatively similar and, at the same time, quite different from the distribution of K^+^. Quantitatively, there is still a three-fold difference between the distribution coefficients for Li^+^ and Na^+^ when the Na/K ATPase pump is active. The concentration of Li^+^ in the medium slightly influences the [Li]_i_/[Li]_o_ and [Na]_i_/[Na]_o_ ratios. The coefficient of Li^/^Na discrimination, *c*_*d*_, better characterizes the differences between the distributions of Li^+^ and Na^+^ because it is not affected by possible errors in determination of the cell water content; *c*_*d*_ increases slightly but significantly, when [Li]_o_ increases from 1 to 10 mM. The value of *c*_*d*_ ≈ 3 in U937 cells is within the range found for human erythrocytes at very low (0.1–1.0 μM) Li^+^ concentrations in the plasma [29], for patients regularly taking Li^+^ when its concentration in the plasma of about 0.5–1 mM [14] and for the muscle tissue of various marine invertebrates (2–5) and fishes (3) at natural [Li]_o_ [30–32]. Evidently, the Li^+^–Na^+^ discrimination is determined by very conservative factors.

**Table 4:** Balanced Li^+^ and Na^+^ distribution across the cell membrane in U937 cells incubated for 4 h in RPMI with addition of 1–10 mM LiCl.

[Li]_o_ mM	Li^+^_i_	Na^+^_i_	Li^+^_i_/[Li]_o_	Na^+^_i_/[Na]_o_	*c*_*d*_	P	[Li]_i_/[Li]_o_	[Na]_i_/[Na]_o_	*n*
	μmol·g^-1^		μmol·g^-1^·mM^-1^						
1	4.6 ± 0.5	190 ± 17	4.29 ± 0.40	1.59 ± 0.16	2.76 ± 0.12	P(1–10) = 0.002	0.82 ± 0.07	0.30 ± 0.03	10
2	9.6 ± 1.1	203 ± 24	5.00 ± 0.63	1.70 ± 0.22	2.93 ± 0.15	P(2–10) = 0.007	0.97 ± 0.11	0.32 ± 0.04	9
5	22.6 ± 2.3	175 ± 18	4.44 ± 0.37	1.48 ± 0.17	3.23 ± 0.07	P(1–5) = 0.017	0.85 ± 0.06	0.28 ± 0.03	7
10	47.8 ± 3.2	174 ± 17	5.02 ± 0.40	1.45 ±0.15	3.58 ± 0.10	P(5–10) = 0.02	0.96 ± 0.06	0.28 ± 0.03	9
5+Oua	42.3 ± 1.6	824 ± 61	7.61 ± 0.43	7.03 ± 0.38	1.08 ± 0.06		1.46 ± 0.08	1.35 ± 0.07	6

The Li/Na discrimination coefficients, *c*_*d*_ = ([Li]_i_/[Na]_i_)·([Na]_o_/[Li]_o_), were determined as the ratio of Li^+^ and Na^+^ concentrations assayed in the same samples of cell TCA extracts to those in the medium. Statistical significance P of the differences in *c*_*d*_ was calculated for the indicated pairs and for data collected on the same days. Incubations with 10 μM ouabain and 5 mM LiCl were carried out for 12 h. Means ± SE for *n* determinations are given.

Table 5 shows balanced distributions of Li^+^ and Na^+^ at indicated [Li]_o_ calculated at constant parameters fitted to the balanced state at [Li]_o_ = 5 mM (Table 2). The computation yields a 10% decrease in *c*_*d*_ as [Li]_o_ changes from 1 to 10 mM; in reality, however, *c*_*d*_ increases by about 30%. Table 6A shows how *c*_*d*_ depends on the parameters *ilc*, *pl*, *kp* and *alpha*. Computed values would match the experimental data if *ilc* or *kp* varied with [Li] _o_ as shown in Table 6B. The general conclusion is that the model based on constant parameters predicts the general trends in Li^+^ distribution reasonably well; nevertheless, further improvements achieved by fine-tuning of the parameters suggest that they vary slightly with [Li]_o_. We also conclude that the maintenance of the balanced [Li]_i_ ≈ 0.9[Li]_o_, as observed in the experiments, can indeed be provided by an outward net Li^+^ flux due to Li/Na coupled transfer, LN. If LC were primarily responsible for Li^+^ maintenance then the net Li^+^ flux would have the opposite sign, i.e. directed inward, down the Li^+^ electrochemical gradient. The Li^+^ and Na^+^ net fluxes via the LN pathway are smaller than the active Na^+^ flux through the pump by a factor of 18 (Table 3). Thus, only a minor fraction of the Na/K ATPase energy consumption in this example falls on the secondary active transport of Li^+^. The inward Li^+^ net flux of 0.0839 down the gradient splits up between the LC pathway (62%) and the channels (38%).

**Table 5:** Observed and computed Li/Na discrimination coefficient *c*_*d*_ = ([Li]_i_/ [Na]_i_)·([Na]_o_/[Li]_o_) in U937 cells under the balanced state at different [Li]_o_.

[Li]_o_ mM	*c*_*d*_		Computed values		
	observed	computed	[Li]_i_ mM	[Li]_i_ /[Li]_o_	[Na]_i_ mM
1	2.76 ± 0.12	3.31	0.883	0.88	37.3
2	2.93 ± 0.15	3.27	1.763	0.88	37.7
5	3.23 ± 0.07	3.17	4.375	0.88	38.6
10	3.58 ± 0.10	3.02	8.660	0.87	40.1

Parameters used in the computation were as specified in Table 2; *kv* was corrected for the particular values of [Li]_o_

**Table 6:** Effects of *alpha*, *kp*, *pl* or *ilc* on the computed Li/Na discrimination coefficient *c*_*d*_ under the balanced state at variable [Li]_o_.

**A** *c*_*d*_ computed at different but constant parameters; other parameters are as in Table 2					
[Li]_o_ mM	*Alpha* 0÷0.001÷0.003	*Kp* 0.00015÷0.00025		*Pl* 0.002÷0.005	*Ilc* 0.0001÷0.0003
1	3.31 ÷ 3.24 ÷ 3.09	3.79 ÷ 2.98		3.06 ÷ 3.56	2.82 ÷ 3.84
2	3.27 ÷ 3.20 ÷ 3.06	3.75 ÷ 2.94		3.03 ÷ 3.52	2.80 ÷ 3.79
5	3.17 ÷ 3.11 ÷ 2.97	3.63 ÷ 2.85		2.95 ÷ 3.40	2.75 ÷ 3.61
10	3.02 ÷ 2.98 ÷ 2.85	3.45 ÷ 2.73		2.83 ÷ 3.21	2.66 ÷ 3.37
**B** *c*_*d*_ observed and computed at *ilc* and *kp* that vary in parallel with [Li]_o_					
[Li]_o_ mM	*c*_*d*_ observed	*c*_*d*_ computed			
		*ilc* variable*kp* = 0.0002		*kp* variable*ilc* = 0.00018	
		*ilc*	*c*_*d*_	*kp*	*c*_*d*_
1	2.76 ± 0.12	0.00009	2.74	0.00029	2.76
2	2.93 ± 0.15	0.00012	2.93	0.00025	2.94
5	3.23 ± 0.07	0.0002	3.26	0.00019	3.25
10	3.58 ± 0.10	0.00042	3.57	0.00014	3.56

### Validation of the model by studying ion balance transients caused by blocking the pump with ouabain or replacement of external Na^+^ for Li^+^

**Fig 4:**
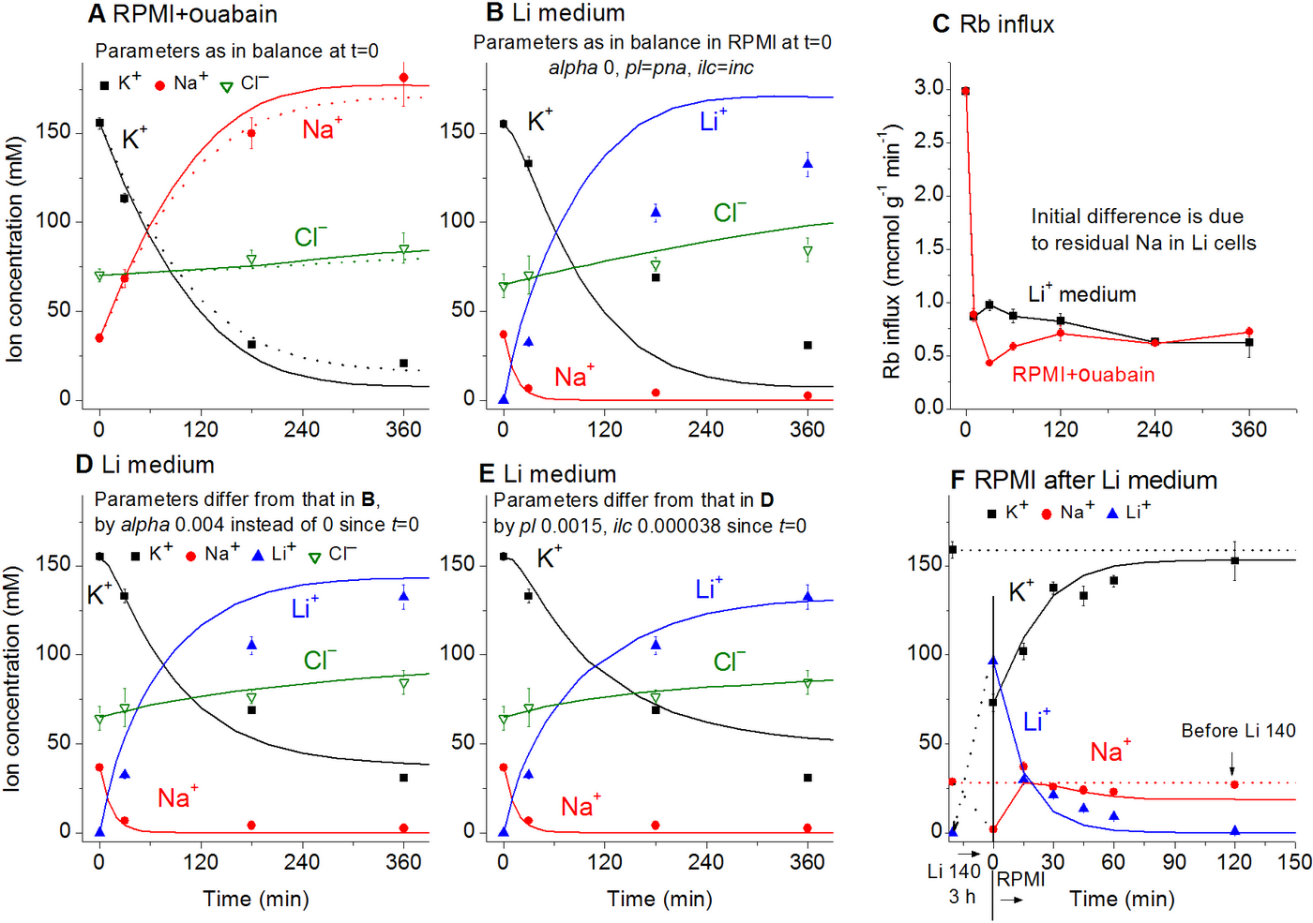
Ion changes caused by blocking the pump with ouabain or replacement of external Na^+^ for Li^+^. Symbols–experimental data obtained in U937 cells (means ± SE, n = 4), solid lines–calculated data (with the exception of C). (A) Ouabain was added at *t* = 0. Calculations were run for the same parameters as in the balanced state at *t* = 0: *na0* 140, *k0* 5.8, *cl0* 116, *B0* 48.2, *kv* 1, *na* 35, *k* 156, *cl* 70, *beta* 0, *gamma* 1.5, *pna* 0.00301, *pk* 0.023, *pcl* 0.00405, *inc* 3.4E-5, *ikc* = *inkcc* = 0, *hp* 400. Before blocking the pump, *beta* was 0.039. Dotted lines were obtained for the same parameters except *beta* was changed from 0 to 0.001. (B) The parameters employed in the calculation were the same as at the balanced state in a Na medium, i.e. *na0* 0.01, *k0* 5.5, *cl0* 147, *l* 140, *B0* 17.5, *kv* 1, *na* 37, *k* 155, *l* 0.0001, *cl* 65, *alpha* 0, *beta* 0.039, *gamma* 1.5, *pna* = *pl* = 0.00353, *pk* 0.023, *pcl* 0.00413, *inc* = *ilc* 3E-5, *ikc* = *inkcc* = *ilkcc* = 0, *kp* 0.0002, *hp* 400. (C) Rb^+^ influx in cells placed at t = 0 into a Li^+^ medium or into RPMI with 0.1 mM ouabain. Rb^+^ influx was measured over 10 min at each given time point. (D) Parameters as in B except *alpha* = 0.004. (E) Parameters as in *B* except *alpha* = 0.004, *pl* = 0.0015 and *ilc* = 3.8E-5. (F) Transient processes following replacement of external Li^+^ back for Na^+^. The cells were preincubated in the Li medium for 3 h, then returned to RPMI at *t* = 0. Calculation parameters: *na0* 140, *k0* 5.8, *cl0* 116, *l* 0.001, *B0* 48.2, *kv* 1, *na* 2, *k* 73, *l* 96, *cl* 76, *alpha* 0.04, *beta* 0.08, *gamma* 1.5, *pna* = *pl* = 0.00353, *pk* 0.023, *pcl* 0.00413, *inc* = *ilc* 3E-5, *ikc* = *inkcc* = *ilkcc* = 0, *kp* 0.0002, *hp* 150.

The dynamics of Li^+^ gain and K^+^ exit following a complete external Na^+^ for Li^+^ substitution resembles the case when the pump is inhibited by ouabain (Fig 4A and 4B). As soon as intracellular Na^+^ is lost, the Na/K-ATPase pump stops (Fig 4C). Therefore, in cells with low internal Na^+^, 80% of K^+^ is replaced with Li^+^ by 4 h. In spite of this, the cells maintain their size and water content at nearly the same level as in the control [10]. The normal K/Na ratio can be restored by returning cells to RPMI medium (Fig 4F).

The data on transients caused by substitution of Na^+^ for Li^+^, as well as by application of ouabain, support the validity of the model in general. A good match between the observed and predicted dynamics of ion disturbance following ouabain treatment has been reported previously [9]; the more detailed analysis presented here shows that further improvement can be achieved by assuming a small (2.5%) residual activity of the pump (the dotted line in Fig 4A). The match between the theory and the Li^+^ experiment is less perfect (Fig 4B); nevertheless, the reciprocal equivalent changes in intracellular Li^+^ and K^+^ concentration are predicted sufficiently well. The fit is improved if one assumes a Li/K pump activity with the rate coefficient 0.004, i.e. at 10% of the normal Na/K pump activity (Fig 4D). The other way to improve the fit is to decrease *pl* by a factor 2.3 and to increase *ilc* by a factor 1.3 (Fig 4E). Varying the other parameters does not improve the agreement between the measured and computed data. The best fit of the transient kinetics following reverse replacement of external Li^+^ for Na^+^ is achieved by assuming activation of the Li/K and Na/K pumps (alpha = 0.04 and beta = 0.08, Fig 4F). Comparison of the calculated and experimental data is the way to find out how ion-transporting mechanisms are altered under variable conditions. Note that the rate of the transient process associated with the macroscopic changes in cell ion content may be much lower than the rate of the tracer ^22^Na^+^ or ^36^Cl^−^equilibration alone due to rapid Na/^22^Na or Cl/^36^Cl exchange (compare Figs 1 and 4).

### Li/Na exchange with and without specific Li/Na countertransporter

**Fig 5:**
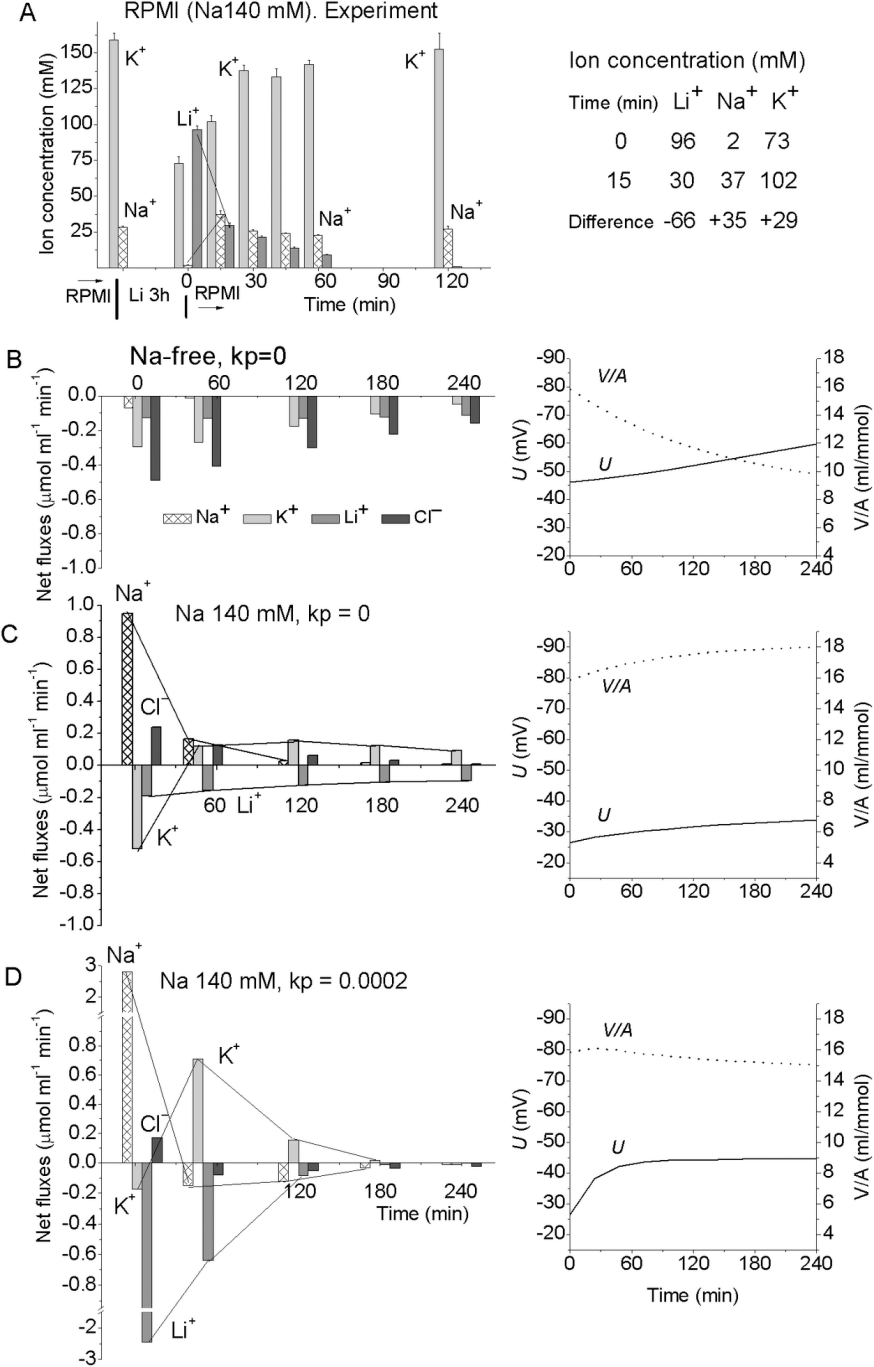
Recovery of ion balance in U937 cells preloaded with Li^+^. (A) Observed changes in [Li]_i_, [K]_i_, [Na]_i_ after placing cells into Li^+^-free RPMI medium. Computed net fluxes in the model cells placed in a Li^+^-free medium without (B) or with (C and D) Na^+^, as well as without (B and C) or with (D) Li/Na countertransporter. The parameters used in the calculations: *kv* 1, *na* 2, *k* 73, *l* 96, *cl* 76, *alpha* 0, *beta* 0.039, *gamma* 1.5, *pna* 0.00349, *pk* 0.0229, *pl* 0.00349, *pcl* 0.00426, *inc* 3E-5, *ilc* 0, *ikc* = *inkcc* = *ilkcc* = 0, *hp* 400. (B) *na0* 0.01, *k0* 5.8, *cl0* 116, *l0* 0.01, *B0* 188.2; (C, D) *na0* 140, *k0* 5.8, *cl0* 116, *l0* 0.01, *B0* 48.2; *kp* is indicated on the graphs. The graphs on the right show the changes in membrane potential and water balance.

The Li/Na exchange is usually evaluated by comparison of Li^+^ efflux from cells loaded with Li^+^ into the media with or without Na^+^. Computational modeling allows separating the effect of the specific Li/Na countertransport and the effect of the mandatory electroneutrality of ion transfer. Fig 5 shows the changes in Li^+^, Na^+^, K^+^ and Cl^−^concentrations in U937 cells preloaded with Li^+^ during recovery of the ion balance in Li^+^-free media with and without Na^+^. Experimental results are compared to the calculations based on the models with or without Li/Na countertransporter. When cells are placed in RPMI half of the Li^+^ exit during the first 15 min is balanced by Na^+^ gain, whereas the other half is balanced by K^+^ gain. Later a decrease in both in Li^+^ and Na^+^ concentrations takes place, which is balanced by accumulation of K^+^. Both experiment and computation show that Li^+^ exit into a Na^+^-containing medium during the first stage is balanced to a large extent by Na^+^ gain even in the absence of Li/Na countertransport (*kp* = 0) while later it is balanced by K^+^ gain. By contrast, Li^+^ exit into a Na^+^-free medium is accompanied by K^+^ exit and the loss of both cations is compensated by an equivalent Cl^−^loss. The difference in the recovery kinetics in these cases is associated with different changes in the membrane potential and water balance (Fig 5, graphs on the right). Li^+^ loss and Na^+^ gain are enhanced in the model with a Li/Na countertransporter (*kp* = 0.0002) but only during the first stage, when the outward Li^+^ net flux is counterbalanced by an inward Na^+^ net flux. These complicated relationships between ion fluxes should be taken into account when Li/Na substitution is used to test for alteration in a specific Li/Na countertransporter.

### Full list of major unidirectional fluxes across the plasma membrane in U937 cells under the balanced state. Coupled Na/Na and Cl/Cl exchange

Once validated, the model can help estimate every unidirectional and “electroneutral” coupled flux. Table 7 shows unidirectional fluxes through the pump, the channels and the NC, LC, and LN transporters in U937 cells balanced in RPMI+5 mM LiCl. Summation of all unidirectional effluxes or influxes gives the overall flux representing continuous traffic of ions into and out of cell, the “turnover” flux. This flux determines the rate of equilibration of ions-tracers. The computed Li^+^ entire flux of 0.1849 μmol·min^-1^· (ml cell water)^-1^ at [Li]_i_ = 4.4 mM corresponds sufficiently well with the observed equilibration rate coefficient of 0.042 min^-1^ at [Li]_o_ = 5 mM (Fig 3A and 3B). The sum of the computed unidirectional Na^+^ influxes through the channels, NC and LN (or the sum of effluxes through the pump, channels, NC, and LN) is significantly less than the observed turnover flux, which is about 5.8 μmol·min^-1^·(ml cell water)^-1^ (Fig 3C). Therefore, there has to be one more unaccounted for Na^+^ flux. It is the equivalent Na/Na exchange, which has no impact on ion concentrations or on membrane potential but adds significantly to the turnover flux.

**Table 7:** The computed net and unidirectional Na^+^, Li^+^, and Cl^−^fluxes via major routes and the turnover fluxes in U937 cells equilibrated in RPMI+5 mM LiCl.

Ion	[Ion]_i_ mM	Flux	PUMP	Channels	NC	LC	LN	Turnover flux	Na/Na	Cl/Cl
Na^+^	38.6	Net	-1.5051	0.9975	0.4248	0	0.0839			
		Influx	0	1.0462	0.5082	0	0.1225	5.8	4.14	
		Efflux	-1.5051	-0.0486	-0.0834	0	-0.0386	5.8	4.14	
Li^+^	4.38	Net	0	0.0319	0	0.0521	-0.0839			
		Influx	0	0.0374	0	0.1089	0.0386	0.185		
		Efflux	0	-0.0055	0	-0.0568	-0.1225	0.185		
Cl^–^	72.1	Net	0	-0.4712	0.4248	0.0521	0			
		Influx	0	0.1861	0.5082	0.1089	0	10.18		9.3
		Efflux	0	-0.6573	-0.0834	-0.0568	0	10.18		9.3

Parameters used in the computation as in Table 3; the fluxes in μmol·min^-1^· (ml cell water)^-1^

**Fig 6:**
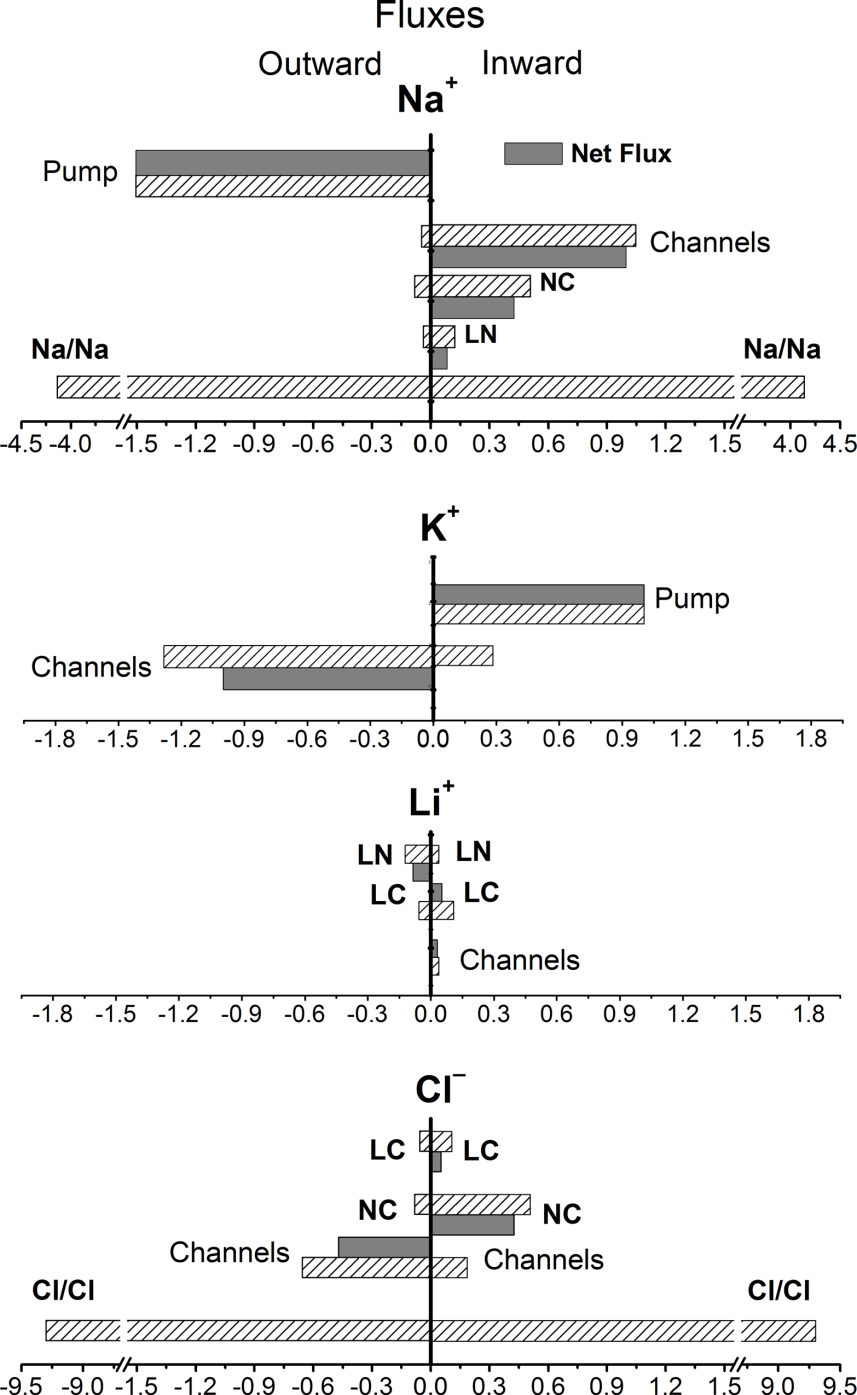
Computed net and unidirectional Na^+^, K^+^, Li^+^, and Cl^−^fluxes via major pathways in cells equilibrated in RPMI+5 mM LiCl. The parameters used in the computation are as in Table 3; the flux units are defined in Table 1.

In order to find the rate coefficient for the Na/Na coupled exchange, the ^22^Na gain or exit was computed by using Eq (9) for Li^+^ with parameters similar to those of the Na^+^ carrier. A Li^+^-free media was assumed in this case and the Li/Na coupled exchange had the meaning of ^22^Na/Na exchange, while the parameter *kpNa* fitting the experimental ^22^Na curve represented the rate coefficient for the ^22^Na/Na exchange. The curves 1 and 2 in Fig 3C show the computed kinetics of ^22^Na exit for *kpNa* = 0 (no Na/Na exchange) and *kpNa* = 0.0008, respectively. As the cells cannot distinguish between ^22^Na and Na, the rate coefficients *kp* for both isotopes should be equal. Therefore, the macroscopic flux related to the Na/Na coupled exchange in the normal Li^+^-free media according to Eq (10) is *J*_*NN*_ = *kpNa*·[Na]_o_·[Na]_i_ = 0.0008·140·37 = 4.14 μmol·min^-1^·(ml cell water)^-1^. Interestingly, the rate coefficient *kpNa* is four times larger than the coefficient *kp* for the Li/Na exchange. The Na/Na coupled exchange appears to be very large in comparison with the fluxes via other pathways (Fig 6). It exceeds the pump-mediated Na^+^ efflux by a factor of 2.8, the channel Na^+^ influx by a factor of 4, and accounts for 71% of the total Na^+^ flux. Therefore Na^+^ influx involved in secondary active Li^+^ efflux relates to active Na^+^ efflux by the Na/K-ATPase pump as 0.1225 to 1.5051 (at 5 mM external Li^+^). We may conclude that Li^+^ active transport in other human cells at therapeutic Li^+^ concentration does not load significantly the sodium pump. A major mechanism for Li^+^ efflux appears to be the Li/Na exchange whereas for Li^+^ influx it is the LC cotransport.

It should be noted that the current concept of ion transfer via channels and transporters assumes that there are forward and backward fluxes in every pathway, including electroconductive channels. Evidently, we should distinguish the genuine coupled exchange and “phenomenological” self-exchange which is determined by reversibility of transport process irrespective of its molecular mechanism. In the used formalism, the difference between direct and reverse fluxes is much greater for channels than for transporters. There is much evidence that Li/Na exchange may occur via various pathways where sodium-linked transport takes place, e.g. NHE [11, 33, 34], NKCC [13], NCC [35], Na^+^ coupled phosphate transporter [12, 36, 37] etc. Similar transporters may be responsible also for specific Na/Na exchange.

The K^+^ flux balance in the minimal cell model is relatively simple, because the impact of fluxes via NKCC and KC cotransporters in U937 cells should be small due to small forces driving the ions by these mechanisms. The sum of K^+^ fluxes via the pump and channels fully corresponds to the measured total K^+^ turnover flux and there is no need to hypothesize a K/K equivalent exchange. The coupled Cl/Cl exchange has been described in various cell types [38–41]. The approach developed in the present study allows computation of fluxes due to Cl/Cl equivalent exchange along with other unidirectional Cl^−^fluxes although. However, the executable file should be slightly modified. That would go beyond the scope of the present paper; nevertheless, we have included in Fig 6 our unpublished experimental and computational results on Cl/Cl exchange in U937 cells. It accounts for 91% of the total Cl^−^flux in U937 cells under considered conditions.

## Conclusion

Monovalent ion fluxes across the plasma membrane are not independent, but linked through the factors that can be classified into three groups. First, fluxes are constrained by the conditions of osmotic balance and electroneutrality. Second, they are linked through coupled ion transfer, such as occurs in the Na/K-ATPase pump and various co- and counter-transporters. Third, transporters can share common dependence on the same factors, such as pH (as is the case with Na/H and Cl/HCO_3_ countertransporters) or on mediators of intracellular signaling: such dependence can manifest itself in variable rate coefficients. The factors of the first two groups can be accounted for by mathematical modeling and numerical integration of the flux balance equations. This has been demonstrated in a series of fundamental works from several research groups [3, 5, 6, 9, 25, 42–44]. Unfortunately, most experimentalists continue to neglect computational approaches because modeling includes many parameters whose evaluation is difficult and unreliable and because of the scarcity of software available for researchers with limited programming expertise. We thus attempted to develop such a computational tool. Following the previous authors [24–26, 28] we used a mathematical model of ion traffic across the cell membrane that differentiates ion transfer systems by a thermodynamic characteristic, i.e. by the driving force acting on ions. Each pathway can be characterized by a single rate coefficient that can be rigorously determined in the experiment and used as an intrinsic physiological parameter of a resting cell. These resting values can then be applied to a system where the balance has been disturbed to see if the model continues to accurately describe cell behavior. If it does, that can be taken as an indication that the parameters have not changed.

We applied such an approach to an experiment where all the external sodium was replaced with lithium. Our study shows that the analysis of the balanced state allows a rather accurate prediction of the following: (1) changes in the internal Li^+^ concentration under variation of external Li^+^ within 1–10 mM; (2) kinetics of the transients caused by blocking the pump with ouabain. The latter result exceeded expectations because ouabain treatment causes profound changes in the cell ion content, and one could expect that the properties of transporters and channels would be affected as well. Nevertheless, the parameters remained unchanged. Not so good was the match between the computed and measured kinetics after full replacement of external Na^+^ for Li^+^. However, the general time course of changes in ion and water content and the reciprocal changes in intracellular K^+^ and Li^+^ were as predicted. In interpreting the results of numerical modeling, one should keep in mind that the main limiting factor in solving physiological problems is the accuracy of experimental data, and not the fine details of the model.

The experimental data necessary for rigorous determination of the parameters for U937 cells under the balanced state include monovalent ion concentrations, cell water content, ouabain-sensitive and -resistant Rb^+^ influx and the rate coefficients of equilibration of ^22^Na, ^36^Cl and Li^+^ at low concentrations. The NKCC and KC transporters were not included in the model because of their low expression in U937 cells. However, our software allows their inclusion in the model if desired. For determination of the NKCC and KC rate coefficients, additional information would be needed. That information could be obtained, for instance, from measurements of the bumetanide- and DIOA-sensitive portions of the Rb^+^ influx. Thus, the developed computational approach can be applied to any cell types.

The developed software enables one to estimate quantities that are difficult to measure directly, i.e. unidirectional fluxes, one-to-one coupled ion exchange, transmembrane electrochemical and electric potential differences, and the rate coefficients for ion transfer via specific pathways. Furthermore, it makes it possible to distinguish between the relatively simple effects caused by linking of fluxes due to the basic conditions of osmotic balance and electroneutrality and more complex effects caused by alteration of ion pathways per se, i.e. the rate coefficients of ion transfer. We believe that this software can be useful both in research (for example, for finding conditions where the effects of alteration of specific pathways on measurable characteristics are most pronounced) and in education, for demonstration of regulation of cell water, membrane potential and electrochemical gradients by individual channels and transporters.

## Appendix

### Equations

The mandatory conditions of macroscopic electroneutrality and osmotic balance can be written when Li^+^ is presented in the media or in cell as:

$${\left[Na\right]}_{i}+{\left[K\right]}_{i}+{\left[Li\right]}_{i}-{\left[Cl\right]}_{i}+\frac{zA}{V}=0$$ (1)

$${\left[Na\right]}_{o}+{\left[K\right]}_{o}+{\left[Li\right]}_{o}+{\left[Cl\right]}_{o}+{\left[B\right]}_{o}={\left[Na\right]}_{i}+{\left[K\right]}_{i}+{\left[Li\right]}_{i}+{\left[Cl\right]}_{i}+\frac{A}{V}$$ (2)

The subscripts *i* and *o* indicate the intracellular and extracellular concentrations in mM, respectively;

*V* is the volume occupied by intracellular water (ml), which assumed approximately equal to the cell volume; *A* is the total amount (in mmol) of membrane-impermeant osmolytes with mean valence z; [*B*]_*o*_ is the external concentration of the membrane-impermeant solute (see Table 1 for symbols and definitions).

From Eqs 1 and 2 it follows that

$$z=({\left[Cl\right]}_{i}-{\left[Na\right]}_{i}-{\left[K\right]}_{i}-{\left[Li\right]}_{i})/({\left[Na\right]}_{o}+{\left[K\right]}_{o}+{\left[Li\right]}_{o}+{\left[Cl\right]}_{o}+{\left[B\right]}_{o}-{\left[Cl\right]}_{i}-{\left[Na\right]}_{i}-{\left[K\right]}_{i}-{\left[Li\right]}_{i})$$ (3)

$$V/A=1/({\left[Na\right]}_{o}+{\left[K\right]}_{o}+{\left[Li\right]}_{o}+{\left[Cl\right]}_{o}+{\left[B\right]}_{o}-{\left[Cl\right]}_{i}-{\left[Na\right]}_{i}-{\left[K\right]}_{i}-{\left[Li\right]}_{i})$$ (4)

$$V=zA/({\left[Cl\right]}_{i}-{\left[Na\right]}_{i}-{\left[K\right]}_{i}-{\left[Li\right]}_{i})$$ (5)

Dependences of ion fluxes via electroconductive channels and NKCC, KC, NC cotransporters and the pump on ion concentrations were formulated by the simplest way ignoring saturation phenomena [3–6, 25]. The equation for Li^+^ fluxes was similar to that for Na^+^, but without pump component. The Li/Na countertransport added which was formulated like the Na/H countertransport in [6].

To express the fluxes, the following equations used:

$$\frac{d({\left[Na\right]}_{i}V)}{dt}=V\{p_{Na}u({\left[Na\right]}_{i}\;\text{exp}(u)-{\left[Na\right]}_{o})/g-\beta {\left[Na\right]}_{i}+J_{NC}+J_{NKCC}+J_{LN}\}$$ (6)

$$\frac{d({\left[K\right]}_{i}V)}{dt}=V\{p_{k}u({\left[K\right]}_{i}\;\text{exp}(u)-{\left[K\right]}_{o})/g+\beta {\left[Na\right]}_{i}/\gamma +\alpha {\left[Li\right]}_{i}/\gamma +J_{NKCC}+J_{LKCC}+J_{KC}\}$$ (7)

$$\frac{d({\left[Cl\right]}_{i}V)}{dt}=V\{p_{Cl}u({\left[Cl\right]}_{o}\;\text{exp}(u)-{\left[Cl\right]}_{i})/g+J_{NC}+J_{KC}+2J_{NKCC}+J_{LC}+2J_{LKCC}\}$$ (8)

$$\frac{d({\left[Li\right]}_{i}V)}{dt}=V\{p_{L}u({\left[Li\right]}_{i}\;\text{exp}(u)-{\left[Li\right]}_{o})/g-\alpha {\left[Li\right]}_{i}+J_{LC}+J_{LKCC}-J_{LN}\}$$ (9)

Here *u* is the dimensionless membrane potential related to the absolute values of membrane potential *U* (mV) as *U* = *u*RT/F = 26.7*u* for 37°C and *g* = 1 − exp(*u*).

The left-hand sides of Eqs 6–9 represent the rates of change in the cell ion content. The right-hand sides express net fluxes via channels, the Na/K pump (*β*[Na]_i_, *β*[Na]_i_/*γ*), the Li/K pump (*α*[Li]_i_, *α*[Li]_i_/*γ*), cotransporters NC, KC, LC, NKCC, LKCC (*J*_*NC*_, *J*_*KC*_, *J*_*LC*_, *J*_*NKCC*_, *J*_*NKCC*_), and countertransport Li/Na (*J*_*LN*_). The rate coefficients *p*_Na_, *p*_K_, *p*_L_, *p*_Cl_ characterizing channel ion transfer are similar to Goldman’s coefficients. Fluxes *J*_*NC*_, *J*_KC,_ *J*_KC,_ *J*_*NKCC*_, *J*_*LKCC*_ and *J*_*LN*_ are related to intracellular and external ion concentrations as:

$$\begin{array}{l} J_{NC}=i_{NC}({\left[Na\right]}_{o}{\left[Cl\right]}_{o}-{\left[Na\right]}_{i}{\left[Cl\right]}_{i})\\ J_{KC}=i_{KC}({\left[K\right]}_{o}{\left[Cl\right]}_{o}-{\left[K\right]}_{i}{\left[Cl\right]}_{i})\\ J_{LC}=i_{LC}({\left[Li\right]}_{o}{\left[Cl\right]}_{o}-{\left[Li\right]}_{i}{\left[Cl\right]}_{i})\\ J_{NKCC}=i_{NKCC}({\left[Na\right]}_{o}{\left[K\right]}_{o}{\left[Cl\right]}_{o}{\left[Cl\right]}_{o}-{\left[Na\right]}_{i}{\left[K\right]}_{i}{\left[Cl\right]}_{i}{\left[Cl\right]}_{i})\\ J_{LKCC}=i_{LKCC}({\left[Li\right]}_{o}{\left[K\right]}_{o}{\left[Cl\right]}_{o}{\left[Cl\right]}_{o}-{\left[Li\right]}_{i}{\left[K\right]}_{i}{\left[Cl\right]}_{i}{\left[Cl\right]}_{i})\\ J_{LN}=k_{P}({\left[Na\right]}_{o}{\left[Li\right]}_{i}-{\left[Na\right]}_{i}{\left[Li\right]}_{o}) \end{array}$$ (10)

Here *i*_NC_, *i*_KC_, *i*_LC_, *i*_NKCC_, *i*_LKCC_, and *k*_*P*_ are the rate coefficients.

By multiplying expression 1 by ***V*** and taking a time derivative, we obtain the following relationship:

$$\frac{d({\left[Na\right]}_{i}V)}{dt}+\frac{d({\left[K\right]}_{i}V)}{dt}+\frac{d({\left[Li\right]}_{i}V)}{dt}-\frac{d({\left[Cl\right]}_{i}V)}{dt}=0,$$ (11)

From Eqs 6–9 and considering Eq 11, a transcendental equation for potential *u* follows:

$$F(u)=\text{exp}(u)-\frac{p_{Na}{\left[Na\right]}_{o}+p_{K}{\left[K\right]}_{o}+p_{L}{\left[Li\right]}_{o}+p_{Cl}{\left[Cl\right]}_{i}+\beta {\left[Na\right]}_{i}(1-1/\gamma )/u+\alpha {\left[Li\right]}_{i}(1-1/\gamma )/u}{p_{Na}{\left[Na\right]}_{i}+p_{K}{\left[K\right]}_{i}+p_{L}{\left[Li\right]}_{i}+p_{Cl}{\left[Cl\right]}_{o}+\beta {\left[Na\right]}_{i}(1-1/\gamma )/u+\alpha {\left[Li\right]}_{i}(1-1/\gamma )/u}=0$$ (12)

### Algorithm

Modeling of the flux balance in a cell requires a numerical solution of a Cauchy problem for the set of Eqs 6–9 [9, 45]. The values of *z* and *V* compatible with the conditions of electroneutrality and osmotic balance have to be computed at the outset. Cell volume *V* and potential *u* are found at every step of numerical integration. The transcendental Eq 12 is solved in two stages. First, one finds the values of *u* that ensure that *F*(*u*) crosses zero at physically meaningful values of *u*. Next, the process of root refining ZEROIN from the calculation package FMM [46] is applied. Numerical integration of 6–9 can be accomplished by the explicit Euler method. To monitor the approach to stationary state, time derivatives of sodium, potassium and chloride concentrations are displayed.

### Executable file

This system of equations does not have a physically meaningful solution for every value of parameters or initial concentrations. This was taken into account in program development, and the following diagnostic tools were provided to assist the user:

1 In studying the dependence of the process on external ion concentrations, one enters new concentrations along with parameter *kv* = *S*_*oN*_ / *S*_*oO*_, where the second subscripts stand for the old (*О*) and new (*N*) values.

$$S_{oO}={\left[Na\right]}_{oO}+{\left[K\right]}_{oO}+{\left[Li\right]}_{oO}+{\left[Cl\right]}_{o\;O}+{\left[B\right]}_{oO}$$

$$S_{oN}={\left[Na\right]}_{oN}+{\left[K\right]}_{oN}+{\left[Li\right]}_{oN}+{\left[Cl\right]}_{o\;N}+{\left[B\right]}_{oN}$$

If the parameter *kv* or the initial concentrations do not meet the requirements of electric or osmotic equilibrium, a message “BAD INITIAL DATA” is generated.

2 The program finds potential *u* in the voltage range +5 mV to –173 mV as sufficient to the possible physiological values and then stops. The negative limit may be changed if necessary. If *u* is not found because of unrealistic parameters or initial concentrations, the program reports "RANGE LIMIT”.
3 If, in the process of computation, the internal sodium concentration drops below 0.1, a message “LOW SODIUM” appears, and the computation stops.

The executable file LIP, as well as the files: How to use LIP.doc, DATAP.txt, LIP.txt, and RESP control.txt is included in the zipped file LIP.zip available at www.cytspb.rssi.ru/lab_vereninov/LIP.zip. The source code LIP-pas.zip can be obtained upon request from verenino@gmail.com, via1938@mail.ru, v.yurinskaya@mail.ru. It can be freely reproduced and distributed with explicit reference to or acknowledgment of the material author.
